# The Effects of Several Natural Protoberberine Alkaloids and Cinnamic Acid Derivatives Used for Traditional Medicine on the Membrane Boundary Potential and Lipid Packing Stress

**DOI:** 10.3390/ijms262211237

**Published:** 2025-11-20

**Authors:** Svetlana S. Efimova, Polina D. Zlodeeva, Quan Minh Pham, Huong Thi Thu Trinh, Ha Minh Le, Van Thị Hong Nguyen, Long Quoc Pham, Olga S. Ostroumova

**Affiliations:** 1 Institute of Cytology of Russian Academy of Science, Tikhoretsky Ave. 4, St. Petersburg 194064, Russia; efimova@incras.ru (S.S.E.);; 2 Institute of Chemistry, Vietnamese Academic of Science and Technology (ICH, VAST), 18 Hoang Quoc Viet, Hanoi 113000, Vietnam; 3 Laboratory of Biophysics, Institute for Advanced Study in Technology, Ton Duc Thang University, 19 Nguyen Huu Tho Street, Ho Chi Minh City 700000, Vietnam; 4 Faculty of Pharmacy, Ton Duc Thang University, 19 Nguyen Huu Tho Street, Ho Chi Minh City 700000, Vietnam

**Keywords:** protoberberine alkaloids, berberine, tetrahydropalmatine, nitidine, lipid bilayers, coumarins, cinnamic acid, osthole, membrane boundary potential, lipid packing and ordering

## Abstract

Here we elucidated the effects of natural protoberberine alkaloids (rotundine, berberine, and nitidine) and cinnamic acid derivatives (ethyl-4-methoxycinnamate and osthole) found in Vietnamese medicinal plants, on the boundary potential of lipid bilayers and phase behavior of membrane lipids. Lipid bilayers were composed of neutral phosphatidylcholines (PC) and negatively charged phosphatidylserines (PS). Tested compounds did not produce any noticeable changes in the boundary potential with the exception of osthole, which caused a potential drop by about 30 mV independently of the membrane phospholipid composition. Protoberberine alkaloids did not demonstrate an ability to greatly influence phase transition of PC, while they dramatically disturbed PS melting by integrating two different lipid states by merging the low-melting component into the higher one. Ethyl-4-methoxycinnamate and osthole were able to decrease the temperature and sharpness of the PC and PS phase transition, although the effect on PS was higher. We also revealed that ethyl-4-methoxycinnamate and osthole diminished the melting point of both components of PS transition without the changes in their relative impacts. The observed membrane activity of the tested compounds may be related to their physiological and pharmacological potential.

## 1. Introduction

Vietnam has a long tradition of traditional medicine, using natural active ingredients from plants to care for community health. Through recent scientific research, a series of specific active ingredients from plant materials have been extracted, purified, and clarified their chemical structures, as well as their interesting physiological activities. Natural active ingredients used as drugs or simulated carriers for semi-synthetic drugs are the current trend in the world compared to fully synthetic active ingredients because of their friendliness, ease of tolerance, as well as safety, and few side effects on living organisms [1].

Natural active substances with the nature of small molecules will have an advantage in circulating through the cell membrane; however, understanding the process of permeation of these active substances, as well as their effects on the lipid matrix, is an issue that needs to be clarified, which will help to direct research to enhance the effectiveness of drugs, including the related problem of drug resistance of potential future drugs.

The natural protoberberine alkaloids and derivatives of cinnamic acid found in Vietnamese medicinal plants exert notable bioactive effects. In particular, rotundine, l-tetrahydropalmatine, is an isoquinoline alkaloid primarily isolated from the roots of *Stephania rotunda* (*Menispermaceae* family). Traditionally used in Chinese and Vietnamese medicine for its sedative, analgesic, and antispasmodic effects, rotundine modulates dopaminergic and serotonergic neurotransmission underlying its sedative and anxiolytic properties. Additionally, it shows anti-inflammatory, antioxidant, and cardioprotective effects, indicating broader therapeutic potential. Due to diverse bioactivities and low toxicity, rotundine is gaining attention as a promising lead for drug development in pain, addiction, and neurodegenerative disease treatment [2,3,4].

Berberine is a natural isoquinoline alkaloid, isolated from *Coscinium fenestratum*, with broad pharmacological effects, including antimicrobial, anti-inflammatory, antioxidant, antidiabetic, lipid-lowering, anticancer, and neuroprotective properties. Berberine demonstrates significant antimicrobial and antifungal activities, notably against fungal pathogens like *Penicillium digitatum*. It is able to disrupt microbial membranes and inhibit nucleic acid synthesis. Berberine reduces inflammation by suppressing key signaling pathways. These combined actions make berberine a promising candidate to combat antibiotic resistance and chronic inflammation. It also shows strong cytotoxic effect on various melanoma cancer cells [5,6,7,8,9,10].

Nitidine is a natural benzophenanthridine alkaloid derived from *Zanthoxylum myriacanthum*. It exhibits significant anticancer properties by inhibiting cell proliferation, inducing apoptosis, and causing cell cycle arrest across various cancer types including breast, glioma, colorectal, lung, and kidney cancers. Nitidine modulates critical signaling and lysosome-dependent pathways, which are involved in tumor growth and survival, and demonstrates synergistic effects with chemotherapy agents like doxorubicin. It notably suppresses cancer stemness markers, reducing self-renewal capacity of cancer stem cells. These properties position nitidine as a promising compound for cancer treatment due to its multi-targeted mechanisms and ability to inhibit metastasis and stem cell–like traits in tumors [11,12,13,14,15].

Ethyl 4-methoxycinnamate, a key compound found in *Kaempferia galanga*, exhibits diverse biological activities including anti-inflammatory, analgesic, antioxidant, antimicrobial, antispasmodic, and UV-protective effects. It exerts significant anti-inflammatory and analgesic actions by inhibiting pro-inflammatory mediators. Its analgesic effect involves modulation of peripheral and central pain pathways, reducing nociceptive responses. Additionally, ethyl 4-methoxycinnamate shows strong antimicrobial activity against various bacteria and fungi. Due to these properties, along with low toxicity and natural origin, it is a promising candidate for developing new treatments targeting inflammatory and pain-related conditions as well as microbial infections [16,17,18].

Osthole is a natural coumarin derivative primarily found in *Cnidium monnieri* and several other medicinal plants. It exhibits a wide range of pharmacological activities, notably anti-inflammatory, antioxidant, anticancer, neuroprotective, hepatoprotective, and osteogenic effects. The anti-inflammatory action of osthole is mainly mediated by modulation of key signaling pathways and inhibition of the expression of pro-inflammatory mediators. Beyond reducing inflammation, its antioxidant effects also help attenuate oxidative stress-related damage. Osthole is considered as a promising natural compound with a favorable safety profile and significant anti-inflammatory and anticancer activity [19,20,21,22,23].

A brief description of the natural sources, chemical structures, and the spectrum of biological activity of the above-mentioned protoberberine alkaloids (rotundine, berberine, and nitidine) and cinnamic acid derivatives (ethyl-4-methoxycinnamate and osthole) are given in Table 1.

Due to the variety of biological activities and the lipophilic structure of protoberberine alkaloids and cinnamic acid derivatives, one can suppose that these molecules can influence cell metabolism by affecting membranes. In favor of this assumption, it is known that the compounds summarized in Table 1 are modulators of the activity of various ion transport systems. In particular, rotundine acts on Kv1.5 channels, acid-sensing ion channels, L-type calcium channels, ATP-sensitive potassium channels, α7 and α4β2 nicotinic acetylcholine receptors, and NMDA receptors [24,25,26,27,28,29,30]. Berberine targets L- and T-type calcium channels, KCNH6 channels, hERG channels, basolateral KCNQ1 channels, Kir2.1 channels, Ca^2+^ release-activated Ca^2+^ channels, ATP-sensitive K+ channels, TRPV1, TRPV3, and TRPV4 channels, volume-sensitive chloride channels, and α7 nicotinic acetylcholine receptors [31,32,33,34,35,36,37,38,39,40,41]. The effects of nitidine on myocardial electronic activity by enhancing current through the potassium channels are documented [42]. The literature findings provided wide evidence for modulating effects of osthole on α receptors, TRPV3, voltage-gated Na^+^ channels, TRPA1, GABA receptors, and N-and P/Q-type Ca^2+^ channels [43,44,45,46,47,48,49]. Moreover, osthole also shifts the voltage dependence of the inactivation curve of Ca(v)1.2 channels to more negative potentials, potentiates ΔF508-CFTR chloride channel gating, and inhibits voltage-dependent L-type Ca^2+^ channels [50,51,52]. The wide diversity of ion channels regulated by protoberberine alkaloids and derivatives of cinnamic acid may indicate the significant role of compound-induced alteration in the lipid microenvironment. Among the direct evidence, Errico et al. [53] demonstrated the berberine-induced increase in fluorescence anisotropy of 1-(4-trimethylammoniumphenyl)-6-phenyl-1,3,5-hexatriene p-toluenesulfonate that indicated a higher degree of lipid packing in membranes of SH-SY5Y cells in the presence of the alkaloid. Molecular dynamics simulations performed by Dhakal et al. [54] revealed dipalmitoylphosphatidylcholine interdigitation at the low molar ratio of berberine to lipid.

Biological membranes are complex multimolecular systems comprising various components, which can make analyzing and interpreting experimental data especially difficult. To overcome these challenges, utilizing model lipid systems, like planar lipid bilayers, is preferred. Such models provide a superior level of precision and allow for selective control over experimental conditions. The aim of the current study was to characterize quantitatively the changes in physical properties of model membranes of various lipid compositions treated with protoberberine alkaloids (rotundine, berberine, and nitidine) and cinnamic acid derivatives (ethyl-4-methoxycinnamate and osthole). Electrophysiological methods, as the most sensitive, were used to study the influence of the tested compounds on the transmembrane distribution of electrical potential, and calorimetric study was performed to describe the effects on the membrane packing density by observing the phase transitions of lipids in the presence of additives. Obtaining information on the effect of tested compounds on neutral and negatively charged model membranes allows us to evaluate the lipid selectivity and predict the corresponding pharmacological potential.

## 2. Results and Discussion

### 2.1. Membrane Boundary Potential

**Figure 1:**
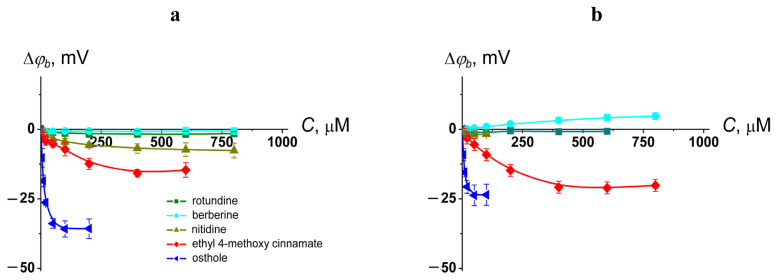
Dependences of the changes in the membrane boundary potential (∆*φ_b_*) on the concentration (*C*) of alkaloids (rotundine, berberine, and nitidine) and cinnamic acid derivatives (ethyl-4-methoxycinnamate and osthole) in the membrane bathing solution. The membranes were composed of DOPC (**a**) and DOPS (**b**) and bathed in 0.1 M KCl (pH 7.4). Transmembrane potential was equal to 50 mV. The relation between the color of symbol and the agent type is given on the panel (**a**).

The influence of natural protoberberine alkaloids (rotundine, berberine, and nitidine) and derivatives of cinnamic acid (ethyl-4-methoxycinnamate and osthole) on the membrane boundary potential (*φ_b_*) was studied. Figure 1 shows the dependences of agent-induced changes in *φ_b_* of DOPC bilayers on the concentration (*C*) of tested compounds. All curves are characterized by saturation, with the maximum changes in the *φ_b_* at an infinitely high concentration of the agent (Δ*φ_b_*(*max*)). Table 2 demonstrates the mean values of Δ*φ_b_*(*max*). One can see that only osthole was characterized by an ability to significantly decrease *φ_b_* of DOPC membranes (by about 35 mV). The highest value of dipole moment (µ) of the osthole molecule (Table 2) might indicate the predominant role of the dipole component in the osthole-induced changes in *φ_b_*. Ethyl-4-methoxycinnamate decreased *φ_b_* by about 15 mV, while rotundine, berberine, and nitidine did not affect the boundary potential of DOPC bilayers (Figure 1a, Table 1). With the replacement of neutral DOPC for negatively charged DOPS, nitidine lost its ability to slightly reduce *φ_b_*, while berberine acquired the ability to insignificantly increase *φ_b_* of DOPS membranes. This tendency might be explained by a positive charge of molecules of these protoberberine alkaloids. Ethyl-4-methoxycinnamate and osthole did not demonstrate noticeable lipid specificity of their potential-modifying action (Figure 1b, Table 2).

The membrane boundary potential is composed of two components: the surface potential, which arises from the charges of membrane-forming lipids and adsorbed molecules, and the dipole potential, which is linked to the orientation and hydration state of the polar heads of lipid molecules [55,56]. Amphiphilic molecules adsorbed onto or intercalating within the membrane matrix can alter the electrical potential gradient through their charges and intrinsic dipole moments or by modifying the hydration dynamics of lipids [57,58]. Given that certain berberine and nitidine molecules exist in the cationic form at physiological pH (7.4), one can hypostatize the increase in *φ_b_* by impacting the surface component. However, no significant changes were observed for either neutral or negatively charged membranes, despite electrostatic attraction between the negatively charged bilayer and the cationic forms of these compounds. This indicates that berberine and nitidine, including their charged forms, reduce the dipole component of the boundary potential. Probably, the presence of four methyl groups flanking the molecule influences the orientation of rotundine dipole moment within the membrane, aligning it more parallel to the membrane surface diminishing possible effect of this neutral alkaloid on bilayer dipole potential. This fact is in agreement with the critical role of both the magnitude and orientation of the dipole moment in small molecule–membrane interactions [59].

### 2.2. Lipid Melting

**Figure 2:**
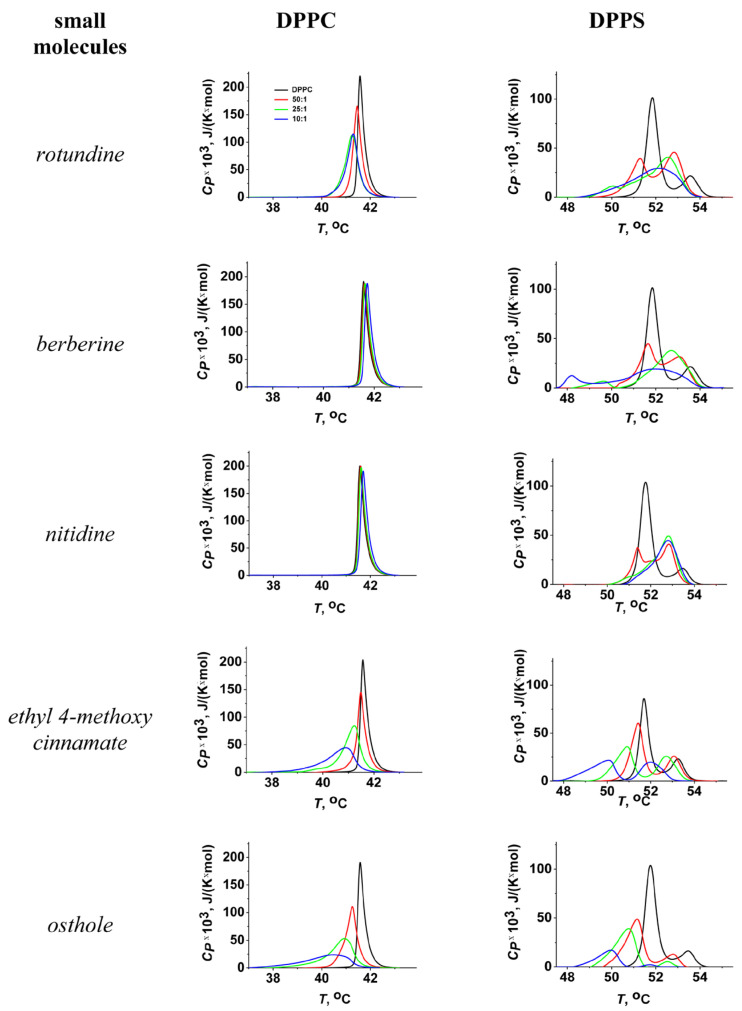
Heating thermograms of DPPC (left column) and DPPS (right column) liposomes in the absence (control, black lines) and presence of alkaloids (rotundine, berberine, and nitidine) and cinnamic acid derivatives (ethyl-4-methoxycinnamate and osthole) at lipid:compound ratio of 50:1 (red lines), 25:1 (green lines), and 10:1 (blue lines).

**Figure 3:**
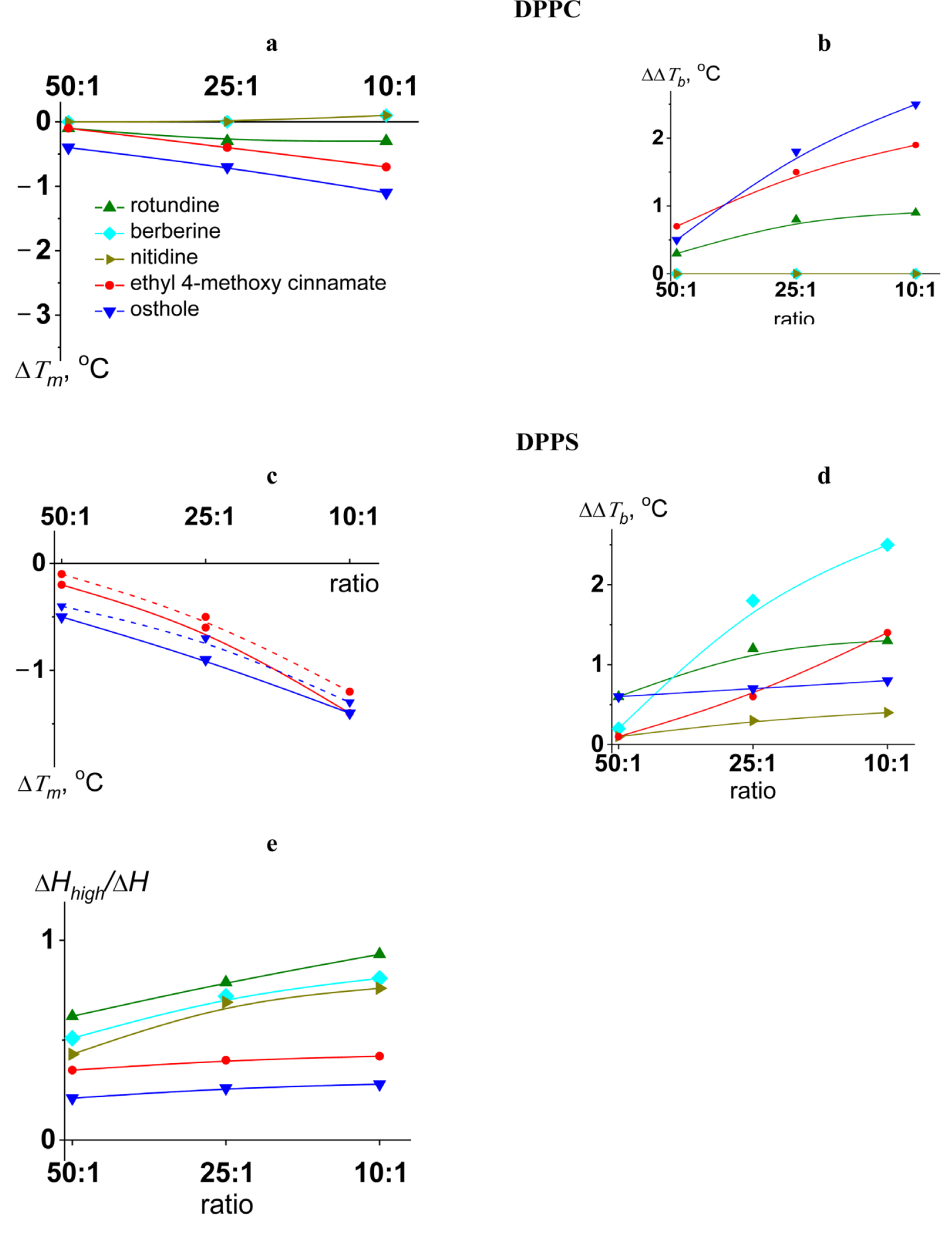
The dependences of the parameters characterizing the thermotropic behavior of DPPC (left column) and DPPS (right column) on lipid:agent molar ratio. The changes in the melting temperature (∆*T_m_*) of DPPC (**a**) and DPPS (**c**) and a width of the peak (∆∆*T_b_*) corresponding to DPPC (**b**) and DPPS (**d**) melting are presented. The deconvolution analysis of the peak related to DPPS melting in the presence of the cinnamic acid derivatives (ethyl-4-methoxycinnamate and osthole) was performed to obtain the changes in transition temperature of low-melting (solid curve) and high-melting (dashed curve) components (**c**) and the relative impact of the component with higher transition temperature to the overall transition enthalpy (**e**).

The transition temperature (melting temperature, *T_m_*) and the width of the main peak (Δ*T_b_*) of pure DPPC were equal to 41.5 ± 0.2 and 2.0 ± 0.2 °C, respectively. Figure 2 shows the heating thermograms of DPPC before and after treatment with tested protoberberine alkaloids and cinnamic acid derivatives at various lipid-to-compound molar ratios. Protoberberine alkaloids did not significantly affect phase transition of PC (Figure 2). Among tested alkaloids, only rotundine was able to slightly decrease the melting point of DPPC in a dose-dependent manner (Figure 3a). Ethyl-4-methoxycinnamate and osthole caused a more significant progressive decrease in the temperature and sharpness of the PC phase transition at decreasing derivative-to-lipid molar ratio (Figure 3a,b). The observed decrease in temperature and cooperativity of lipid melting should be interpreted as a disruption of membrane lipid order induced by the incorporation of the compounds into the lipid bilayer, likely resulting in an increased area per lipid molecule (APL).

The effects of all tested compounds on DPPS melting were stronger than on DPPC (Figure 2). A thermogram of pure DPPS exhibited a complex pattern, with a transition peak that could be deconvoluted into two different components with lower and higher melting temperatures of 51.9 ± 0.2 °C and 53.6 ± 0.2 °C, respectively. The relative impact of the component with a higher transition temperature on the overall transition enthalpy was lower than that of the high-melting component and was about 30%. Taking into account that the more acidic form of hydrated DPPS undergoes transition at a higher temperature but with a lower enthalpy change than the DPPS NH_4_^+^ salt [60], the complex thermotropic behavior of DPPS might indicate the presence of two distinct lipid phases corresponding to different dissociation states [61]. The width of the main transition peak of DPPS (Δ*T_b_*) was equal to 4.1 ± 0.3 °C.

Protoberberine alkaloids caused a dose-dependent broadening of the peak corresponding to the main phase transition (Figure 3d) and the gradual unification of the low- and high-melting components, which in some cases was expressed in the appearance of an intermediate component (Figure S1, Supplementary Materials). Observed effects might be attributed to different interactions of protoberberine alkaloids with two DPPS states, making the two phases merge into one. Moreover, the relative contribution of the high-melting component increased with the increasing alkaloid content by merging the lower temperature peak into the higher one (Figure 3e). Taking into account that the dehydration of DPPS results in an increase in the transition temperature of DPPS NH_4_^+^ salt and does not affect the transition temperature of the DPPS acidic form, the observed effects might be rationalized by membrane dehydration in the presence of protoberberine alkaloids. The data were consistent with the results by Errico et al., demonstrating berberine-induced increase in lipid packing in membranes of SH-SY5Y cells [53]. Molecular dynamic simulations also revealed lipid interdigitation at the nonphysiologically low molar ratio of berberine to lipid [54].

Ethyl-4-methoxycinnamate and osthole diminished the melting points of both components of the DPPS transition (Figure 3c) without altering their relative contributions to the transition enthalpy (Figure 3e). In the presence of osthole the decrease in the total transition enthalpy of DPPS was also observed (Figure 2). This might indicate the induction of a nonlamellar lipid phase by mixing with osthole.

The ability of molecules to disorder membrane lipids is primarily related to their lipophilicity, which can be accessed by the octanol/water partition coefficient. The Log*P* values of positively charged berberine and nitidine molecules are negative (Table 1), indicating that these agents are predominantly dissolved in the aqueous phase compared to non-polar solvent, in particular octanol. At the same time, a slight shift in DPPC melting points to the higher temperatures (Figure 2) in the presence of these protoberberine alkaloids at low lipid:compound ratios suggests that the compounds are still adsorbed onto the membrane surface, causing bilayer dehydration. Applying the approach by Ojogun et al. [62], we estimated partitioning between membrane and water solution (Log*P_m/w_*) from the calorimetric data. The results for DPPC and DPPS membranes are summarized in Table S1 (Supplementary Materials). In the presence of the tested agents, for neutral compounds (rotundine, ethyl-4-methoxycinnamate, and osthole), the Log*P_m/w_* values are lower than the predicted Log*P* values (Table 1) and not practically affected by membrane lipid composition. The reason for the discrepancy between Log*P_m/w_* and Log*P* is related to certain molar volume of solute and higher energy required to form a cavity in the structured phospholipid membrane phase to accommodate the solute than bulk organic solvents such as octanol [63]. It is noteworthy that the logarithm of the distribution coefficient between the negatively charged membrane and the aqueous solution for positively charged berberine and nitidine is positive, demonstrating their predominant presence in the DPPS phase in contrast to octanol.

In summary, natural protoberberine alkaloids (rotundine, berberine, and nitidine) and cinnamic acid derivatives (ethyl-4-methoxycinnamate and osthole) exhibit distinct effects on lipid bilayers. Osthole uniquely and significantly decreases the membrane boundary potential of PC membranes, likely due to its high molecular dipole moment, while ethyl-4-methoxycinnamate causes a moderate decrease and protoberberine alkaloids show no effect. Protoberberine alkaloids modulate lipid packing stress in negatively charged PS membranes, inducing phase transition broadening and membrane dehydration effects. In contrast, ethyl-4-methoxycinnamate and osthole disrupt lipid order in both PC and PS bilayers, reducing melting temperature and cooperativity, suggesting increased membrane fluidity and formation of nonlamellar phases, especially with osthole. Overall, the strongest perturbing effect of osthole on membrane boundary potential and lipid phase behavior is potentially underlying its diverse physiological activities. Thus, the found ability of the tested compounds to modify membranes might play a crucial role in delivering pharmacological advantages by changing membrane permeability, fluidity, and the activity of ion channels, and further studies are needed to understand in detail the relationship between membrane activity and the biological action of indicated compounds.

## 3. Materials and Methods

### 3.1. Materials

Nonactin A, KCl, HEPES, pentane, ethanol, and dimethylsulfoxide (DMSO) were purchased from Sigma-Aldrich Company Ltd. (Gillingham, UK). KCl solutions (0.1 M) were buffered using 10 mM HEPES-KOH at pH 7.4.

Lipids, 1,2-dioleoyl-*sn*-glycero-3-phosphocholine (DOPC), 1,2-dioleoyl-*sn*-glycero-3-phosphoserine (DOPS), 1,2-dipalmitoyl-*sn*-glycero-3-phosphocholine (DPPC), and 1,2-dipalmitoyl-*sn*-glycero-3-phospho-L-serine (DPPS) were obtained from Avanti Research (Avanti Research, Inc., Alabaster, AL, USA).

Small natural molecules (rotundine, berberine, nitidine, ethyl 4-methoxy cinnamate, and osthole) isolation is described in [4,15,18,21] and below.

Rotundine isolated from the roots of *Stephania rotunda* was provided by the Natural Compounds and Environmental Protection Laboratory, Institute of Chemistry (Hanoi, Vietnam). Fresh tubers of *Stephania rotunda* were crushed and extracted twice with 4% sulfuric acid solution for 24 h each time. The combined extracts were then neutralized with 10% sodium hydroxide solution to precipitate the alkaloids. The resulting alkaloid precipitate was dried, finely powdered, and extracted with 96% ethanol at 70 °C. The ethanolic extract was concentrated under reduced pressure to obtain crude rotundine. The crude product was further purified by repeated slow recrystallization in 96% ethanol at room temperature to yield pure rotundine as white crystals. The yield of rotundine was 0.32% relative to the weight of the fresh *Stephania rotunda* tubers.

Berberine isolated from *Coscinium fenestratum* was provided by the Natural Compounds and Environmental Protection Laboratory, Institute of Chemistry (Hanoi, Vietnam). The dried powder of *Coscinium fenestratum* stems and roots was macerated with 0.5% sulfuric acid solution for 24 h. Sodium chloride was then added and stirred thoroughly until completely dissolved. The mixture was left to stand for 24 h to allow the precipitation of berberine. The precipitate was separated by filtering and washed to obtain crude berberine chloride. The crude berberine was further purified by reflux extraction with 96% ethanol. The filtrate was left to crystallize overnight at room temperature and then filtered to obtain berberine crystals, which were further purified by repeated slow recrystallization in 96% ethanol. Yellow berberine crystals were obtained from the dried stems and roots of *Coscinium fenestratum*, with a yield of approximately 0.53%.

Nitidine was obtained from the fresh bark of *Zanthoxylum myriacanthum* (9 kg), which was cleaned, cut into small pieces (2–3 cm), and dried at 55 °C. The dried stem bark (4.5 kg) was crushed into a powder, and ethanol was used to extract it three times (each 10 L, 2 days) at 50 °C using a conventional ultrasound-assisted technique, then the solvent was removed *in vacuo* to yield the ethanol extract (250 g). The ethanol extract residue was suspended in water (2.0 L) and successively partitioned with *n*-hexane ethyl acetate (EtOAc) to yield *n*-hexane (80 g) and EtOAc (76 g) residues and a water layer. The water layer fraction was rotary evaporated under vacuum to yield the solid and extract residues. The solid residue was chromatographed on a normal-phase silica gel column, eluted with dichloromethane:methanol:water (10:1:0.05, *v*:*v*:*v*), and then purified again on a reversed-phase silica gel column using methanol:water (3:1, *v*:*v*) as the elution solvent to give compound nitidine.

Ethyl 4-methoxycinnamate isolated from *Kaempferia galanga* was provided by the Natural Compounds and Environmental Protection Laboratory, Institute of Chemistry (Hanoi, Vietnam). *K. galanga* powder (2 kg) was extracted with 96% ethanol (20 L) using an ultrasonic extraction machine at the optimized conditions (extraction power of 100 W, extraction temperature of 56 °C, extraction time of 34 min). The extraction solvent was removed using a vacuum evaporator to yield 500 mL of concentrated extract, which was then placed at 3–5 °C for 20 h. The supernatant was eliminated, and white crystalline ethyl 4-methoxycinnamate (49.8 g) was obtained by recrystallization twice in 96% ethanol.

Osthole isolated from *Cnidium monnieri* was provided by the Natural Compounds and Environmental Protection Laboratory, Institute of Chemistry (Hanoi, Vietnam). A total of 1.5 kg of dried powdered fruits of *Cnidium monnieri* were extracted with *n*-hexane at 50 °C, three times for 4 h each. The combined extracts were concentrated under reduced pressure to remove the solvent, yielding 31.5 g of crude extract. The crude extract was then subjected to normal-phase column chromatography and eluted with a gradient of *n*-hexane:EtOAc (100:0, 60:1, 40:1, 20:1, 5:1, 1:1, 0:100), affording seven fractions (H1–H7). Fraction H4 was further purified by column chromatography using *n*-hexane:EtOAc (10:1) as the eluent, yielding four subfractions (H4.1–H4.4). *Osthole* (0.35 g) was obtained as white crystals from subfraction H4.3 by repeated recrystallization in acetone.

### 3.2. Methods

#### 3.2.1. Measurement of the Membrane Boundary Potential

Planar lipid bilayers were prepared using the Montal and Muller method [64] on an aperture in a 10 μm thick Teflon film that separated two compartments of the Teflon chamber. The aperture, with a diameter of 50 μm, was pretreated with hexadecane. Lipid membranes were made from pure DOPC or DOPS. After the membrane was fully formed and stabilized, stock solutions of nonactin (10 mM in ethanol) were added to both compartments to achieve the final concentrations ranging from 0.1 to 1 μM. The steady-state conductance of K^+^-nonactin was modulated by adding the tested compounds to both sides, from millimolar stock solutions in DMSO, into the membrane-bathing solution (0.1 M KCl, 10 mM HEPES, pH 7.4) to achieve final concentrations ranging from 2.5 μM to 1 mM.

Ag/AgCl electrodes were used to apply voltage and measure transmembrane current. Experiments were conducted using an Axopatch 200B amplifier (Molecular Devices, LLC, Orleans Drive, Sunnyvale, CA, USA) in voltage clamp mode. Data were digitized using Digidata 1440A and analyzed with pClamp 10.0 (Molecular Devices, LLC) and Origin 8.0 (OriginLab Corporation, Northampton, MA, USA). The conductance of the lipid bilayer (*G*) was calculated as the ratio between the transmembrane current and the constant transmembrane voltage (*V* = 50 mV).

Calculations of agent-induced changes in the membrane boundary potential (Δφ_b_) were performed according to [65]:

(1) $$∆\phi_{b}=\frac{kT}{e}ln(\frac{G}{G_{0}})$$

where *G* and *G*_0_ represent the steady-state membrane conductance induced by K^+^-nonactin in the presence and absence of molecules, respectively; *e* is the electron charge; *k* is the Boltzmann constant; and *T* is the temperature in Kelvin. Experiments were conducted at room temperature (25 °C).

The value of Δφ_b_(max) was averaged over 3 to 5 independent experiments and presented as mean ± standard deviation (*p* ≤ 0.05).

#### 3.2.2. Differential Scanning Microcalorimetry

Giant unilamellar vesicles composed of DPPC or DPPS were prepared using the electroformation technique with the Vesicle Prep Pro^®^ system (Nanion Technologies, Munich, Germany) following the standard protocol: 3 V, 10 Hz, 58 min at 55 °C. The resulting liposomal suspension contained 4.0 mM lipids and was buffered with 5 mM HEPES at pH 7.4. Small natural molecules were introduced after the liposome suspension was prepared. The lipid-to-molecule molar ratios used were 50:1, 25:1, and 10:1. The suspension was then subjected to heating and cooling at constant rates of 0.2 °C/min and 0.3 °C/min, respectively, using a µDSC 7EVO microcalorimeter (Setaram, Caluire-et-Cuire, France). The reversibility of the thermal transitions was evaluated by immediately reheating the sample after the cooling phase of the previous scan. The temperature dependence of the excess heat capacity was analyzed using Calisto Processing (Setaram, Caluire-et-Cuire, France). For DPPC, thermograms were characterized by the temperature of the main phase transition (melting), *T_m_*. In the case of DPPS, two components of the main transition with lower and higher melting temperatures (*T_m_low_* and *T_m_high_*, respectively) were analyzed separately. The changes in a width of the main peak (the temperature difference between the upper (onset) and lower (completion) boundary of the phase transition, ∆∆*T_b_*), the enthalpy of the main transition (Δ*H*), and the impact of the component with higher transition temperature on transition enthalpy (Δ*H_high_*/Δ*H*) were also examined.

The values ∆*T_m_*, ∆∆*T_b_*, and Δ*H_high_*/Δ*H* are presented as mean ± standard deviation (*p* ≤ 0.05).

## Figures and Tables

**Table 1:** The natural sources, chemical structure, and biological activity of the tested agents.

No.	Compound	Chemical Structure	Original Resource	Biological Activity, [References]
1	rotundine	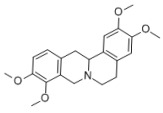	*Stephania glabra (Roxb.) Miers*	sedative, analgesic [2,3,4]
2	berberine	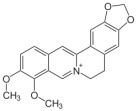	*Coscinium fenestratum*	antibacterial, anti-inflammatory [7,8,9,10]
3	nitidine	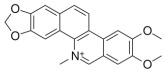	*Zanthoxylum myriacanthum*	cytotoxic activity [11,12,13,14,15]
4	ethyl 4-methoxy cinnamate	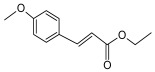	*Kaempferia galanga* L.	anti-inflammatory, analgesic [16,17,18]
5	osthole	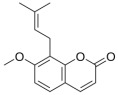	*Cnidium monnieri*	anti-inflammatory [19,20,21]

**Table 2:** The parameters characterizing the ability of protoberberine alkaloids and cinnamic acid derivatives to affect membrane boundary potential of membranes of different lipid composition.

Agent	µ ^$^, D	Log*P* ^&^	∆*φ_b_ (max),* mV	
			DOPC	DOPS
rotundine	2.98	3.32	−2 ± 2	−1 ± 2
berberine	3.35	−0.99	−2 ± 2	5 ± 3
nitidine	2.09	−0.88	−7 ± 3	−2 ± 2
ethyl 4-methoxy cinnamate	2.39	2.65	−16 ± 3	−21 ± 3
osthole	5.81	3.87	−36 ± 2	−24 ± 4

^$^ Calculations of the dipole moments of the molecules (µ) were performed by HyperChem 7.0 (Hypercube, Inc., Gainesville, FL, USA) using the semi-empirical MNDO method; ^&^ calculations of the logarithm of octanol/water partition coefficient (Log*P*) were accomplished by ACD/ChemSketch.

## Data Availability

The original contributions presented in this study are included in the article/Supplementary Materials. Further inquiries can be directed to the corresponding authors.

## Supplementary Materials

The following supporting information can be downloaded at: https://www.mdpi.com/article/10.3390/ijms262211237/s1.
