# Supplementary material for: The Effects of Several Natural Protoberberine Alkaloids and Cinnamic Acid Derivatives Used for Traditional Medicine on the Membrane Boundary Potential and Lipid Packing Stress
Source: Int J Mol Sci. 2025 Nov 20;26(22):11237. doi: 10.3390/ijms262211237 (PMC12653728; doi:10.3390/ijms262211237)
Supplement: Supplementary file 1 [file ijms-26-11237-s001.zip › ijms-3971763-supplementary.pdf]

The effects of several natural protoberberine alkaloids and cinnamic acid derivatives used for traditional medicine on the membrane boundary potential and lipid packing stress

Svetlana S. Efimova[1], Polina D. Zlodeeva[1], Quan Minh Pham[2], Huong Thi Thu Trinh[2], Ha Minh Le[2], Van Thị Hong Nguyen[2], Long Quoc Pham[3,4,*] and Olga S. Ostroumova[1,*]

[1]Institute of Cytology of Russian Academy of Science, Tikhoretsky ave. 4, 194064 St. Petersburg, Russia
[2]Institute of Chemistry, Vietnamese Academic of Science and Technology (ICH,VAST), 18 Hoang Quoc Viet, 113000 Hanoi, Vietnam
[3]Laboratory of Biophysics, Institute for Advanced Study in Technology, Ton Duc Thang University, 19 Nguyen Huu Tho Street, 700000 Ho Chi Minh City, Vietnam
[4]Faculty of Pharmacy, Ton Duc Thang University, 19 Nguyen Huu Tho Street, 700000 Ho Chi Minh City, Vietnam

*correspondences: phamquoclong@tdtu.edu.vn, ostroumova@incras.ru

Supplementary materials

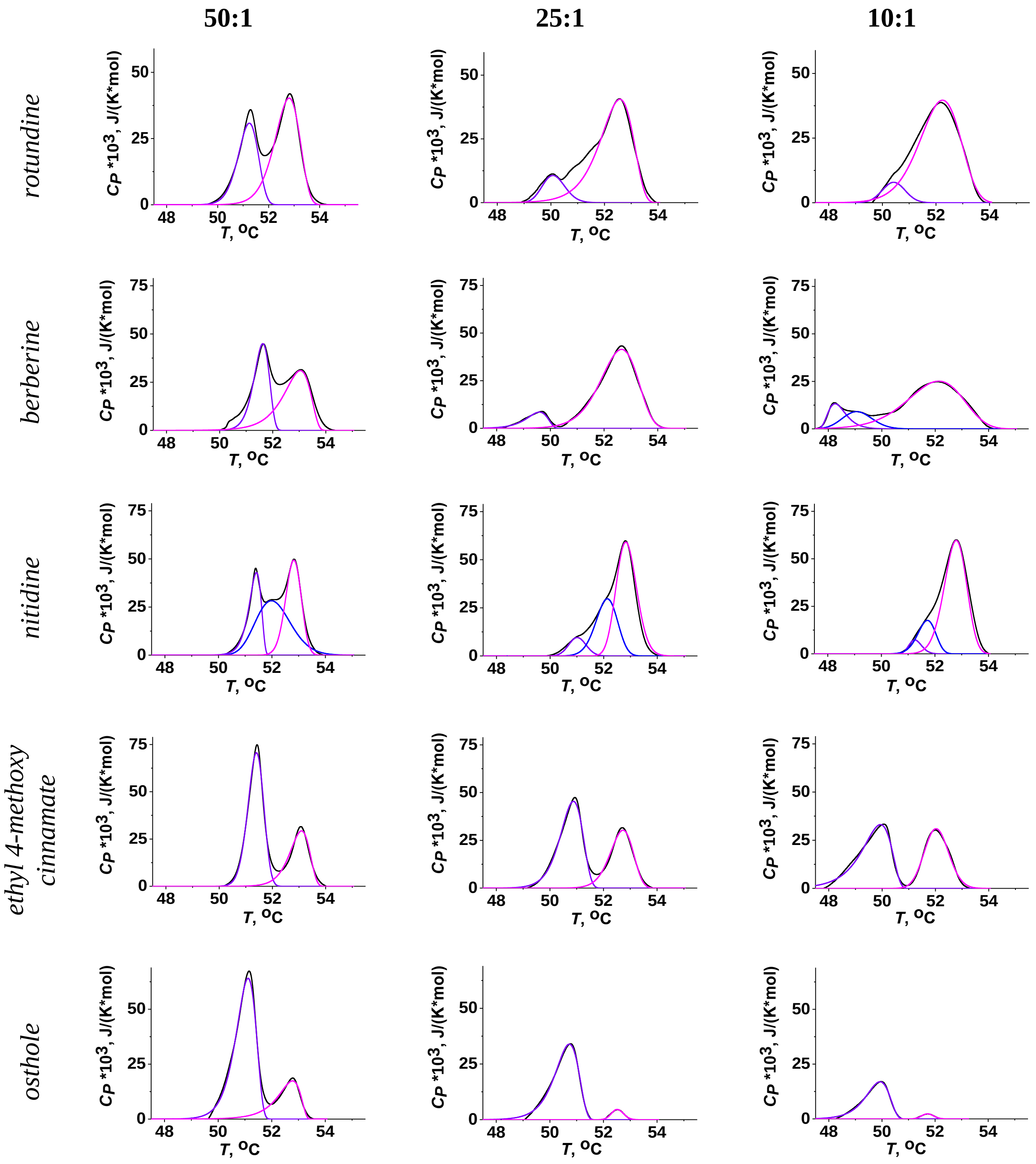

Figure S1. Deconvolution analysis of the main transition peak of DPPS in the presence of alkaloids (rotundine, berberine, and nitidine) and cinnamic acid derivatives (ethyl-4-methoxycinnamate and osthole) at different lipid:agent molar ratio of 50:1, 25:1 and 10:1.

Table S1. The logarithm of membrane/water partition coefficient (Log$P_{m/w}$).

agent	DPPC	DPPS
rotundine	0.78	1.03
berberine	–[#]	1.64
nitidine	–[#]	0.79
ethyl 4-methoxy cinnamate	1.34	1.24
osthole	1.41	1.10

[&]The Log$P_{m/w}$ values were calculated according to [62]. In the case of DPPS the main transition component with lower temperature enriched with tested compound was analyzed.

# – cannot be determined due to the absence of diminishing effect on DPPC melting temperature.
